# The Role of Anthocyanins, Deoxyanthocyanins and Pyranoanthocyanins on the Modulation of Tyrosinase Activity: An In Vitro and In Silico Approach

**DOI:** 10.3390/ijms22126192

**Published:** 2021-06-08

**Authors:** Patrícia Correia, Hélder Oliveira, Paula Araújo, Natércia F. Brás, Ana Rita Pereira, Joana Moreira, Victor de Freitas, Nuno Mateus, Joana Oliveira, Iva Fernandes

**Affiliations:** 1 LAQV-REQUIMTE, Department of Chemistry and Biochemistry, Faculty of Sciences, University of Porto, 4169-007 Porto, Portugal; patricia.correia@fc.up.pt (P.C.); helder.oliveira@fc.up.pt (H.O.); paula.araujo@fc.up.pt (P.A.); nbras@fc.up.pt (N.F.B.); anarita@fc.up.pt (A.R.P.); vfreitas@fc.up.pt (V.d.F.); nbmateus@fc.up.pt (N.M.); 2 Laboratório de Química Orgânica e Farmacêutica, Departamento de Ciências Químicas, Faculty of Pharmacy of the University of Porto, Rua Jorge Viterbo Ferreira nº 228, 4050-313 Porto, Portugal; up201302558@edu.ff.up.pt; 3 Centro Interdisciplinar de Investigação Marinha e Ambiental (CIIMAR), Universidade do Porto, Edifício do Terminal de Cruzeiros do Porto de Leixões, Av. General Norton de Matos s/n, 4050-208 Matosinhos, Portugal

**Keywords:** anthocyanins, deoxyanthocyanins, pyranoanthocyanins, tyrosinase, pigmentation, enzymatic inhibition, molecular docking, structure–activity relationship

## Abstract

Tyrosinase is the central enzyme involved in the highly complex process of melanin formation, catalyzing the rate-limiting steps of this biosynthetic pathway. Due to such a preponderant role, it has become a major target in the treatment of undesired skin pigmentation conditions and also in the prevention of enzymatic food browning. Numerous phenolic-based structures from natural sources have been pointed out as potential tyrosinase inhibitors, including anthocyanins. The aim of the present study was to individually assess the tyrosinase inhibitory activity of eight purified compounds with a variable degree of structural complexity: native anthocyanins, deoxyanthocyanins, and pyranoanthocyanins. The latter two, the groups of anthocyanin-related compounds with enhanced stability, were tested for the first time. Compounds **1** to **4** (luteolinidin, deoxymalvidin, cyanidin-, and malvidin-3-*O*-glucoside) revealed to be the most effective inhibitors, and further kinetic studies suggested their inhibition mechanism to be of a competitive nature. Structure–activity relationships were proposed based on molecular docking studies conducted with mushroom tyrosinase (mTYR) and human tyrosinase-related protein 1 (hTYRP1) crystal structures, providing information about the binding affinity and the different types of interactions established with the enzyme’s active center which corroborated the findings of the inhibition and kinetic studies. Overall, these results support the applicability of these compounds as pigmentation modulators.

## 1. Introduction

The melanin biosynthetic pathway takes place within several distinct species, including bacteria, fungi, plants, and mammals. In humans, the type and degree of pigment production and distribution determines the eye, hair, and constitutive skin color [1,2]. In addition to its coloring effect, the importance of the pigment relies on its capacity to form a supranuclear melanin cap within skin keratinocytes, absorbing UV radiation and dissipating most of the absorbed energy as heat, protecting the skin against UV-induced oxidative stress and cellular damage [3,4]. Despite the acknowledged photoprotective effects of melanin, its aberrant production, secretion, and accumulation is related to several hyperpigmentary skin disorders, such as solar lentigines, melasma, and post-inflammatory hyperpigmentation, while also being reported as a risk factor for melanoma development [5]. The rate of melanogenesis ultimately depends on tyrosinase activity level, which uses its binuclear metal center to catalyze the rate-limiting first two steps of the reaction sequence: the *o*-hydroxylation of monophenols (monophenolase activity) to *o*-diphenols and their subsequent oxidation to *o*-quinones (diphenolase activity) [6,7,8]. Therefore, major efforts have been made in the search for tyrosinase inhibitors, as this has proven to be an effective strategy not only to ameliorate the aesthetic appearance of the skin but also to prevent the undesired enzymatic browning phenomena of food products, which compromises their sensory and nutritional properties, and to date, a myriad of inhibitors have been explored for those purposes. In the context of skin applications, certain conventional lightening agents have long been in the spotlight of controversy: kojic acid, for instance, is associated with possible side effects such as dermatitis and erythema, while hydroquinone has been banned by EU Cosmetic Regulation [8,9]. 

This demand for simultaneously efficacious and well-tolerated agents has been the major driving force of research in this area, and a large number of inhibitors have been identified from natural sources, mostly of phenolic nature, raising increased interest due to their low toxicity [10]. Among the existing subclasses, anthocyanins have been widely reported for their diverse bioactivity spectrum, including their inhibitory effect towards tyrosinase activity, which has been discussed in both contexts of cosmetic/medical and food applications [11,12,13]. Being responsible for the portfolio of vivid colors present in numerous fruits, flowers, vegetables, and leaves, these intriguing compounds are also well-appreciated as natural cosmetic dyes. Structurally, anthocyanins range from simple monoglycosylated units to more complex molecular arrangements involving different degrees of glycosylation and acylation patterns, resulting in an exceptional variety of molecules spread in nature [14]. With respect to their anti-tyrosinase activity, the conducted studies found in the literature are predominantly focused on the evaluation of the effect of extracts from different sources [15,16]. Apart from these complex matrices, a proper evaluation of their individual inhibitory effect is then crucial to understanding their full potential. 

**Figure 1:**
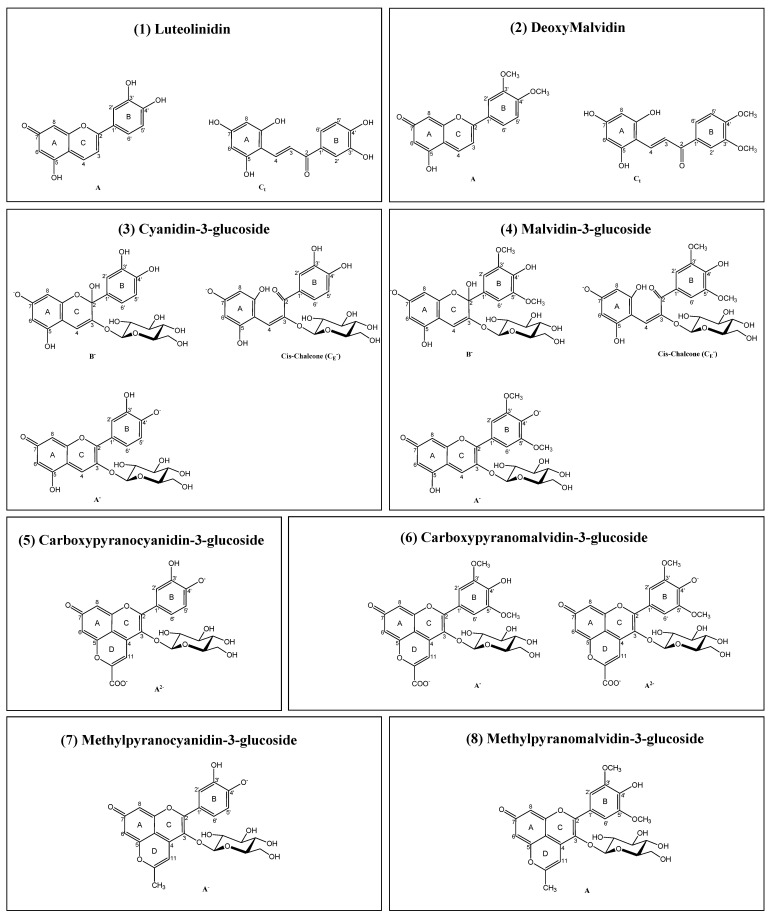
Chemical structures of the main equilibrium forms of the selected group of anthocyanins, deoxyanthocyanins, and pyranoanthocyanins present at pH 7.4. A, quinoidal form; B, hydrated form; C_c_, chalcone cis isomer; C_t_, chalcone trans isomer; and C_E_, chalcone E isomer.

The aim of this study was to individually assess the behavior of 3 specific groups of structurally related compounds on the modulation of tyrosinase activity: (i) cyanidin- and malvidin-3-*O*-glucosides (cy-3-glc and mv-3-glc), particularly abundant in blackberries and in red grapes and respective derived products such as red wines, respectively), (ii) their corresponding carboxy- and methylpyranoanthocyanin derivatives (detected in higher amounts in red wines, juices, and other processed foodstuff), comprising an additional pyranic ring that confers them a higher chromatic stability in a wider pH range due to the absence of hydration reactions, contrary to anthocyanins [17], and (iii) deoxyanthocyanins, luteolinidin, and deoxymalvidin (mainly present in sorghum and sugarcane), which lack the substitution at the C-3 position, a structural feature that also contributes to their greater stability to pH and temperature conditions in comparison with their anthocyanin analogs [18] (Figure 1). A more in-depth analysis regarding their mechanism of action and inhibition mode was also performed by kinetic analysis, along with molecular docking simulations with both mushroom and human constructs. For that purpose, compounds were either isolated from a young red wine or blackberry anthocyanin extract or synthesized, and a structure-activity relationship comprehensive study was performed.

## 2. Results and Discussion

In this study, some representative compounds of three different but structurally related pigments were evaluated for their tyrosinase inhibitory capacity: deoxyanthocyanins, anthocyanins, and pyranoanthocyanins (Figure 1). The different substitution pattern observed in each class has an impact on the spectroscopic properties and polarity of these compounds. Structurally, compounds **3** and **4** differ on the substituents of positions 3’and 5´of ring B. Cyanidin-3-*O*-glucoside (compound **3**) possesses hydroxyl groups on C3´ and a hydrogen on C5´, while in the case of malvidin-3-*O*-glucoside (compound **4**), both C3´ and C5´ have methoxyl groups attached, with compound **4** being less polar than compound **3**. 

In addition to the substitution pattern, other structural features of these compounds also account for a distinguishable physicochemical property: the ability to co-exist in different equilibrium forms depending on pH [19]. This subject is normally disregarded in biological studies, which eventually results in inappropriate interpretations.

Considering that at pH 7.4, pH that normally prevails in the human body, both anthocyanins (compounds **3** and **4**) are present mostly in their hydrated (B) forms and with lower mole fractions in their respective quinoidal (A) and chalcone forms (C_E_ -E isomer); all negatively charged, these were used for the docking studies, using the following abbreviations: 3 to represent cyanidin-3-*O*-glucoside, forms 3-B^−^, 3-A^−^, and 3-C_E_^−^; and 4 to represent malvidin-3-*O*-glucoside, forms 4-B^−^, 4-A^−^ and 4-C_E_^−^. 

From the comparison of compounds **3** and **4** with compounds **5** to **8**, it is possible to observe an extra ring (D ring) that results in a blue shift towards orange wavelengths, 505, 511, 473, and 478 nm, respectively (Figure 1). At pH 7.4, compound **5**, carboxypyranocyanidin-3-*O*-glucoside is almost 100% under the form A^2−^, whilst compound **6**, carboxypyranomalvidin-3-*O*-glucoside, is under the quinoidal form (A) divided into two ionized forms, 6-A^2−^ (60%) and 6-A^−^ (40%) [20,21]. In the case of compound **7**, methylpyranocyanidin-3-*O*-glucoside, and compound **8**, methylpyranomalvidin-3-*O*-glucoside, both are 80% in quinoidal base form, 7-A^−^ and 8-A, respectively. [20,22]. This family of compounds is also less polar than their anthocyanin precursors due to the high number of hydrophobic groups, higher molecular weight, and a reduction on the free hydroxyl groups.

As opposed to anthocyanins, deoxyanthocyanins do not possess either a glucose group or a hydroxyl group in the C ring at C3-position of the flavylium core [23]. This feature makes these pigments much less sensitive to water addition at C2 [24], which reversibly leads to the colorless hemiketal and chalcone forms, and also provides them higher resistance to irreversible chemical degradation. However, these compounds present some limitations such as lower water solubility when compared with water-soluble anthocyanins. Due to their different equilibrium forms, two different structures are present at pH 7.4: for compound **1**, luteolinidin, these were denominated by codes 1-A and 1-C_t_ (chalcone trans isomer), and for compound **2**, deoxymalvidin, the quinoidal neutral base was represented as 2-A and the trans-chalcone as 2-C_t_.

### 2.1. Effect of Anthocyanins and Related Compounds on Tyrosinase Activity

Given the accessibility and commercial availability of mTYR, as opposed to its human analogue, whose large-scale production and purification is still costly and encounters several difficulties, mTYR has been extensively used as a model for identifying potential effective modulators of tyrosinase activity. Despite the structural differences, the active site of tyrosinase comprising two copper ions coordinated by six histidine residues is highly conserved across diverse species [25]. Thus, an initial screening of the **8** compounds consisted of the assessment of their capacity to prevent the oxidation of substrate L-DOPA and consequent conversion to dopachrome. The degree of inhibition of mushroom tyrosinase was found to be considerably different among the tested anthocyanins and derivatives (Table 1).

Non-glycosylated compounds, luteolinidin and deoxymalvidin, had an expressive effect with 58.52% and 57.34% of inhibition, respectively, although less pronounced than kojic acid (76.14%) used as positive control in the conducted experiments. As mentioned earlier, the simple fact that 3-deoxyanthocyanins do not have a hydroxyl group at the C-3 position, thus no acylation or glycosylation substitutions, confers these pigments a greater thermal stability, as well as a higher resistance to pH-induced color loss in mildly to neutral aqueous environments, as their color hues do not vary as much as their anthocyanin analogues, which could be advantageous considering a potential application in cosmetics [18]. In the particular case of luteolinidin, its effect was greater than the anthocyanin analogue, cyanidin-3-*O*-glucoside (compound **3**), with an inhibition of 40.39%. Regarding the methyl- and carboxypyranoanthocyanins (compounds **5–8**), the inclusion of the D-ring clearly affected their capacity to modulate the activity of tyrosinase, with carboxypyanocyanidin-3-*O*-glucoside and carboxypyranomalvidin-3-*O*-glucoside exhibiting a slightly better inhibitory activity than their methylpyranoanthocyanin counterparts. Unlike the methyl group, which unless it finds a hydrophobic pocket to fit in will contribute to the steric hindrance, the carboxyl group in compounds **5** and **6** is negatively charged in biological systems, possibly contributing to stronger electrostatic interactions with the enzyme. Generally, in all pyranoanthocyanins, both glucose and the D-ring may be acting as a bulky group, resulting in a steric hindrance for the functional groups that interact with the active site of the enzyme.

As mentioned earlier, mushroom tyrosinase belongs to the “type 3 copper” family, exhibiting a conserved active site of six histidine residues coordinating the two oxidizing copper ions (CuA and CuB). The diphenolase activity of the enzyme is achieved by the binding of two adjacent hydroxyl groups of the catechol moiety of substrates such as L-DOPA to the copper ions within the active site, which contains a hydrophobic “pocket” sterically favorable to the binding of catechol [26]. In fact, in a previous report, the inhibitory effect of three anthocyanins isolated from seed coats of red and black beans (cyanidin-, delphinidin-, and pelargonidin-3-*O*-glucosides) was compared with their respective aglycons. Although cyanidin-3-*O*-glucoside exhibited the greatest inhibition among the three anthocyanins, which was attributed to the effect of the catechol moiety with 3′, 4′-dihydroxyl groups on the B ring structure for the copper chelation, the effect was less pronounced when compared with its corresponding aglycon structure, cyanidin [27]. In another study, cyanidin-3-*O*-glucoside was the only one showing tyrosinase inhibitory activity among the several flavonoid glycosides tested. Once again, cyanidin, was also tested for comparison and reduced the activity of the enzyme much more efficiently than the glycosylated molecule, reinforcing the weakening effect that the sugar moiety has on the affinity of 3′,4′-dihydroxyphenyl chelating structure to the active site [28].

Similar to the non-glycosylated compounds, malvidin-3-*O*-glucoside (compound **4**) demonstrated an appreciable effect of 54.43% inhibition and, although slightly inferior to the corresponding deoxyanthocyanin, its effect does not appear to be as affected by the presence of the glucose molecule, as in the case of cyanidin-3-*O*-glucoside as discussed earlier. In a previous study, the metal complexing properties of malvidin-3-*O*-glucoside were investigated, and it was suggested that the methoxyl and hydroxyl groups of ring B or the hydroxyl groups at the C5 or C7 position of the molecule could act as the reactive site of the molecule, depending on the pH of the solution [29]. At pH near to 6, the authors suggested the involvement of the methoxyl groups as chelating agents, which may justify the results herein.

### 2.2. Kinetic Analysis and Determination of the Inhibition Type

Considering the appreciable inhibitory effects on tyrosinase activity displayed by luteolinidin, deoxymalvidin, cyanidin-, and malvidin-3-*O*-glucosides, a kinetic study was conducted to determine their mode of inhibition and inhibition parameters. Initial reaction velocity was measured with increasing L-DOPA concentrations, both in the absence and presence of increasing doses of each inhibitor. The collected data were first adjusted to the Michaelis–Menten equation, and the resulting best-fit values for the Michaelis constant (*K*_m_) and maximum enzyme velocity (*V*_max_) were 0.57 mM and 46.4 μM/min, similar to values reported in preceding studies [30].

**Figure 2:**
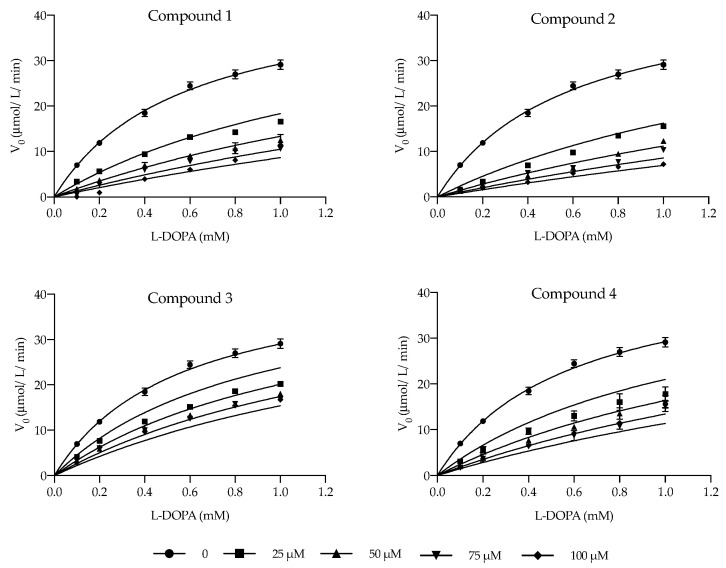
Graphical representation of the enzymatic inhibition behavior displayed by the four analyzed anthocyanin related compounds: compound **1**, luteolinidin; compound **2**, deoxymalvidin; compound **3**, cyanidin-3-*O*-glucoside; compound **4**, malvidin-3-*O*-glucoside. Solid lines represent the optimum global fit of competitive inhibition model to the data. Results are presented as the mean ± standard error deviation (SEM), *n* = 3.

Generally, the mode of inhibition of the so called “true inhibitors” fits into one of these four types of reversible inhibition: competitive (the inhibitor can bind to the free enzyme, competing with the substrate for the same binding site in the active site of the enzyme), uncompetitive (the inhibitor only binds to the enzyme-substrate complex), mixed type (combines both competitive and uncompetitive behavior), and noncompetitive (the inhibitor binds to the free enzyme and an enzyme–substrate complex with the same equilibrium constant) [10]. GraphPad Prism 8.2.1 was used to fit the experimental data to these four models, all of them compared with each other. Akaike’s Information Criteria (AICc) approach was used to compare the fit of competitive, uncompetitive, and noncompetitive models, while extra sum-of-squares *F* test was used to compare the mixed model with the remaining three models, as they are considered to be special cases of the mixed model general equation. The model fitting results consistently suggest that the four compounds exert their inhibitory effect on the diphenolase activity of tyrosinase through a competitive mechanism. Graphical representations of the enzymatic kinetics are exhibited in Figure 2.

Additionally, the mixed model fitting includes an extra parameter, the alpha (α) value, which represents a useful quantitative indicator of the mechanism of inhibition. Essentially, α determines the degree to which the binding of inhibitor alters the affinity of the enzyme for substrate: α > 1 indicates that the inhibitor preferentially binds to the free enzyme, and when that value is very large (α = ∞), the mixed-model approaches the competitive inhibition behavior [31]. A previous study regarding the tyrosinase inhibition kinetics suggested that when α = ∞, the inhibition is purely competitive and indicates that the inhibitor binds to both met (*E*_m_, containing two copper (II) ions) and/or oxy (*E*_o_, containing a peroxo bridge between the two copper (II) ions) forms of the enzyme, whereas 2 < α < 10 denotes a predominantly competitive type of inhibition, where the inhibitor selectively binds to the deoxy form (*E*_d_, containing two copper (I) ions) [32]. In the present experiments, the alpha values obtained from the fitting of mixed model equation were 21.00 and 4.43 for luteolinidin and cyanidin-3-*O*-glucoside, respectively, and there were extremely wide values (∞) in the case of deoxymalvidin and malvidin-3-*O*-glucoside, which further supports the competitive nature of these compounds. This type of inhibition has been reported for phenolic compounds due to their chelating capacity towards the binuclear copper or by structurally mimicking the substrate of the enzyme [28,33]. Cyanidin-3-*O*-glucoside has been described as inhibiting the enzyme competitively and, as mentioned earlier, the docking simulation of the molecule into the catalytic site of tyrosinase proposed that the inhibition was caused by the binding of the 3′,4′-dihydroxyl moiety of the B ring and chelation of the copper ions in the active site, the same structural region attributed to the competitive behavior of quercetin [28,34]. Due to this competitive nature, higher concentrations of substrate will progressively overcome the effect of the inhibitor, as it will ultimately occupy all the binding sites; thus, the *V*_max_ of the enzymatic reaction appears to be unchanged in the presence of increasing compound concentrations, while the apparent *K*_m_ (*K*_m’_) for the substrate increases since a higher concentration of substrate is required to overcome inhibitory effects of the competitor (Table S1, supplementary data).

**Figure 3:**
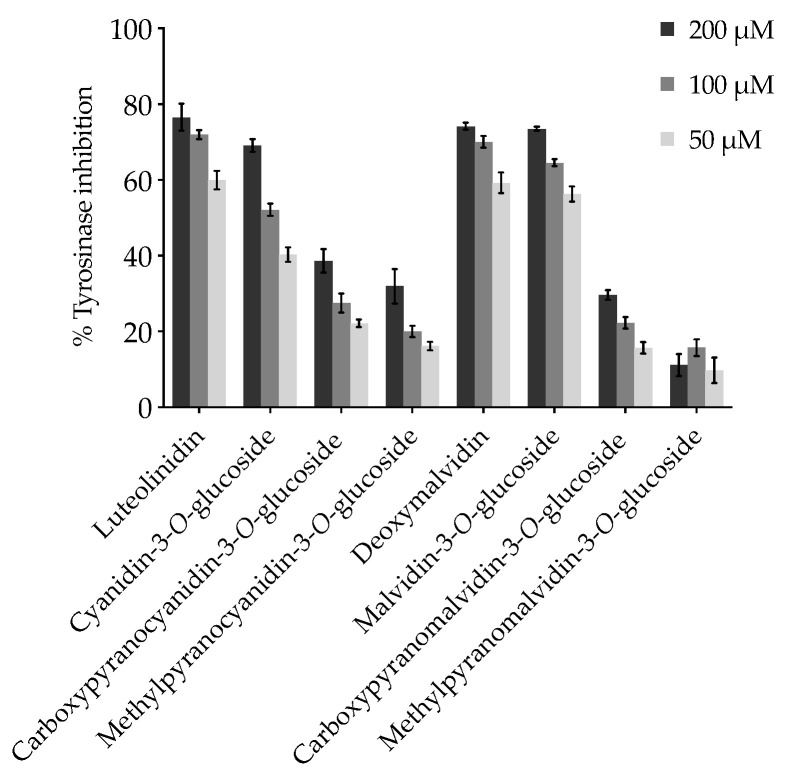
Inhibitory effect of increasing concentrations of the eight tested compounds on L-DOPA oxidation. Results are presented as the arithmetic mean ± standard error deviation (mean ± SEM), *n* = 3.

Inhibition measurements at increasing compound concentrations evidenced that the eight compounds prevent L-DOPA oxidation in a dose-dependent manner (Figure 3), and the IC_50_ values, calculated by non-linear regression analysis, confirmed the potency of luteolinidin and deoxymalvidin as inhibitors, corroborated by their lower inhibition constants, *k*_i_, (Table 2), suggesting a tighter binding of these compounds. On the other hand, their glycosylated analogues, cyanidin- and malvidin-3-*O*-glucosides, exhibited a weaker inhibitory effect given their both higher IC_50_ and *k*_i_ values, particularly in the case of cyanidin-3-*O*-glucoside (IC_50_ = 77.91 μM). Nevertheless, the four compounds considerably inhibited the activity of tyrosinase at 200 μM. Despite the less pronounced effect denoted by pyranoanthocyanins, with carboxypyranocyanidin-3-*O*-glucoside exhibiting the highest inhibition rate (nearly 40% at the concentration of 200 μM) (Figure 3), their greater stability compared with the parent compounds should be highlighted. The protective effect of the additional pyranic ring against the nucleophilic attack of water restricts the formation of hemiketals under weakly acidic to neutral environments, ensuring the stabilization of the color intensity [35], which might represent a beneficial feature considering the potential application of these compounds.

It is important to note that, although the IC_50_ is commonly used to express the potency of a given inhibitor, it is highly dependent on the experimental conditions, such as the source and purity of the enzyme [36], as well as on the substrate fixed concentration, since in the case of competitive inhibition the effectiveness of the inhibitor will progressively weaken as the substrate dose is increased. This explains the discrepancy sometimes observed when attempting to compare reported values in the literature: in the case of cyanidin-3-*O*-glucoside for instance, the IC_50_ of tyrosinase inhibition has been reported in different studies as being 211, 40.3, and 18.1 μM [27,28,37]. Thus, this parameter is more useful from a perspective of relative potency comparison of inhibitors within the same study.

### 2.3. Molecular Docking

The interaction mode of the various pigments into the binding pockets of mTYR and hTYRP1 was also assessed by a molecular docking study. This computational technique has been successfully used to predict the binding of potential inhibitors to tyrosinase enzymes [38,39,40,41,42]. To validate the docking protocol, the crystallographic inhibitor tropolone was re-docked. Figure S1 shows the superposition of both geometries of tropolone (X-ray and top-ranked docking pose) in the active site of mTYR and hTYRP1. Considering the similar position and the small root-mean-square-deviation (RMSD) values between both structures (2.6 Å and 2.2 Å, respectively), our docking parameters fit for describing the correct binding mode of tropolone were deemed. Hence, they were used to evaluate the binding mode of kojic acid (KA), a well-known tyrosinase inhibitor [43], and the various pigments under evaluation. Tables S2 and S3 show the docking results of the different binding poses of each compound in the active sites of mTYR and hTYRP1, respectively, ranked according to the binding energies. There were small energy discrepancies in the binding affinities predicted by docking and the IC_50_ values of all compounds. However, similar differences were also observed in comparable docking studies [38,40,41]. The active sites of mTYR and hTYRP1 enzymes are characterized by a binuclear metal center (Cu and Zn ions, respectively) coordinated by six histidine residues. Previous X-ray data showed that both tropolone and KA bind in the same pocket and their inhibitory effect was attributed to their direct or water-mediated metal-chelating ability and/or to the stacking interaction with one metal-coordinating histidine residue at the active sites [44]. As expected, these interactions were observed in their docking poses, highlighting the importance of the interaction to the metal center. Because of this, and considering the competitive behavior suggested by kinetic studies, the distance between the ligands and the binuclear metal center of mTYR and hTYRP1 was used to rank the docking poses (Table 3; Table 4, respectively). 

**Figure 4:**
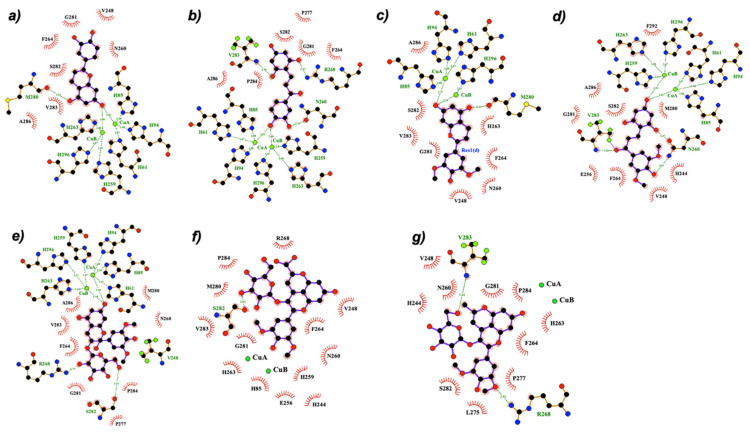
2D representation of the best docking pose of some representative compounds towards the active site of mTYR: (**a**) 1-A, (**b**) 1-Ct, (**c**) 2-A, (**d**) 2-Ct, (**e**) 4-B^−^, (**f**) 6, and (**g**) 8. LigPlot was used for diagram rendering [45]. The 3D poses are depicted in Figure S2.

Figure 4 illustrates the best binding pose of compounds **1**-**A**, **1**-**Ct**, **2**-**A**, **2**-**Ct**, **4**-**B**-, **6**, and **8** against mTYR. Most of the compounds coordinate to the copper center of mTYR and, similar to the KA inhibitor, they coordinate to the metal ions in both bidentated and monodentated forms. However, the native anthocyanins and deoxyanthocyanins (compounds **1** to **4**) interact with smaller or similar distances than the reference standard inhibitors KA and tropolone, whilst carboxy- and methylpyranocyanidin-3-*O*-glucoside (compounds **5** and **6**) have larger Cu-coordinating distances (>3.5 Å). This may explain the better inhibitory activity of the former compounds observed in kinetic experiments. In addition to the metal-coordinating interaction, our results indicate that hydrophobic π–π stacking and dispersive contacts between the compounds and the H263, F264, and V283 residues of mTYR are vital for the binding of these compounds. Hydrogen bonds with N260, H263, and R268 are also relevant, in agreement with previous structure activity relationship (SAR) analysis [40].

Compounds **1–4** can bind towards the Cu ions through the HO-C4′ (B ring) or O-C7 (A ring) groups (exemplified by compound **1** in Figure 4a,b), in accordance with the predicted binding mode of cyanidin-3-*O*-glucoside and morin on tyrosinases [28,46]. However, the latter entrance pose seems to be favored. This probably occurs due to the higher negative charge character of O-C7 as well as by the establishment of an additional H-bond between the HO-C5 (A ring) and the polar side chain of the Cu-coordinated H263, reinforcing the binding to the active site. Furthermore, the binding pocket of mTYR is sterically favorable for accommodating small and aromatic groups such as the AC moiety that mostly covers the active site and is perfectly surrounded by the bulky aromatic and/or hydrophobic residues H263, F264, and V283 (see Figure 4a). The F264 has a key role for the binding of compounds **1–4** because it makes a perpendicular π–π stacking with their AC or B groups and its sidechain seems to act as a lock of the active site, stabilizing the ligand pose and subsequently assisting the interaction to the catalytic metal center. However, the presence of the glucose unit in compounds **3–4** causes a small rearrangement of the AC aromatic plan that slightly perturbs the stacking with both H263 and F264 residues (as seen in the superposition of 2-A, 2-Ct, and 4-B^−^ binding poses in Figure 4). This might justify their lower inhibitory activity on mTYR compared with the corresponding aglycon compounds (**1–2**). Concerning the binding of pyranoanthocyanins (compounds **5–8**), they do not enter so deeply into the active site due to the presence of the bulky pyrano group, and so there is no stacking formation with the H263, which may explain their lower inhibitory activity and highlights the importance of this residue. In fact, the stacking interaction with the homologue residue in hTYRP1 (H381) was suggested as essential and sufficient for binding and tyrosinase inhibition [44]. Instead, the poses of the larger compounds (**5–8**) allow interactions with the residues H244, V248, and S282, justifying the high binding free energies predicted by docking. Additionally, these compounds can enter the active site of mTYR via B ring or pyrano groups (Figure 4e,f), although the B ring is largely favored.

**Figure 5:**
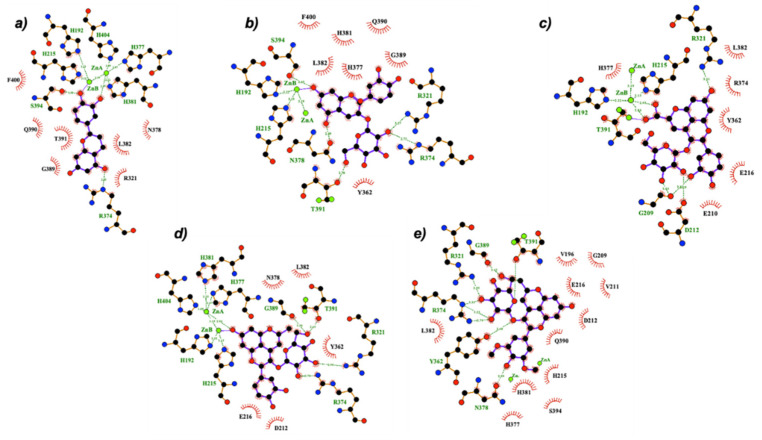
2D representation of the best docking pose of some representative compounds towards the active site of hTYRP1: (**a**) 1-A, (**b**) 3-B^−^, (**c**) 5, (**d**) 7, and (**e**) 8. LigPlot was used for diagram rendering [45]. The 3D poses arthe active sie depicted in Figure S3.

Docking of all compounds against hTYRP1 indicated similar binding modes and interactions to those described for mTYR. Compounds **1–4** and **7** are those with smaller metal-coordinating distances (<2.5 Å). Figure 5 shows the best binding pose of some representative compounds on hTYRP1. Amongst the interaction with Zn cations, they also establish hydrophobic contacts and/or H-bonds with the residues Y362, R374, H381, L382, T391, and S394. The H-bonds and/or stacking with R374, H381, and S394 residues are particularly relevant to strengthening the binding of compounds **1–4**. Indeed, these residues were pointed out as crucial for the binding of standard inhibitors of hTYRP1 [44]. Similar binding mode and interactions were observed for both C_E_- and A^−^ forms of compounds 3–4. However, their respective chalcone forms (C_E_) do not interact with the Y362, H381, and S394 residues. As all forms co-exist in solution, a possible competition between them supports the smaller inhibitory activity of these compounds relative to the respective aglycon molecules. Furthermore, the active site of hTYRP1 does not have any “blocker” residue as does the F264 of mTYR, which may assist the binding of the larger compounds **5–8**. Most pyrano-derivative compounds do not form the stacking and H-bonds with the essential residues H381 and S394 (see Table S3), which once again, may explain their reduced effect observed in inhibition experiments.

The exception is carboxypyrano-mavidin-3-*O*-glucoside (compound **7**) that can enter via the pyrano group (O-C7) in a perpendicular way, promoting vertical π–π stacking with both H381 and Y362 (see Figure 5d). However, this binding pose was not observed for the compound **8** and carboxypyrano derivatives. This could occur due to a favored entrance into the active site by the B ring and carboxylic group, respectively (see Table S3), which reduce (or even abolish) the interactions with the H381 and Y362 aromatic residues (Figure 5c,e).

## 3. Materials and Methods

### 3.1. Isolation and Synthesis of the Different Compounds

Luteolinidin and deoxymalvidin (compounds **1** and **2**, respectively) were synthesized by acidic aldol condensation [47].

Cyanidin- and malvidin-3-*O*-glucosides (compounds **3** and **4**, respectively) were obtained by fractionation of blackberries and young red wine extract, respectively.

Four 3-*O*-glycosilated pyranoanthocyanins were hemi-synthesized: carboxypyranoanthocyanins (compounds **5** and **6**) and methylpyranoanthocyanins (compounds **7** and **8**) [48].

### 3.2. HPLC-DAD and LC-MS Analysis

The synthesized pigments were analyzed by HPLC-DAD using 7.5% formic acid in water (solvent A) and 7.5% formic acid in acetonitrile (solvent B) with a gradient of 3% to 30% of solvent B for 35 min at flow rate of 1.0 mL/min. Column was washed with 100% B and further stabilized in the initial conditions. LC-DAD/ESI-MS analysis of compounds was performed on a Finnigan Surveyor series liquid chromatograph mass spectrometer equipped with a Finnigan LCQ DECA XP MAX (Finnigan Corp., San Jose, CA, USA) mass detector and with an AQUA (Phenomenex, Torance, CA, USA) reversed-phase column (150 × 4.6 mm, 5 μm, C_18_), thermostated at 35 °C. Solvents were A, H_2_O/HCOOH (9.9:0.1), and B, H_2_O/CH_3_CN/HCOOH (6.9:3:0.1). The HPLC gradient used was the same as reported for the HPLC analysis. Double online detection was done in a photodiode spectrophotometer and by mass spectrometry, as previously described [49].

The purity of all compounds was determined by NMR on a Bruker Avance III 600 HD spectrometer (Bruker, Massachusetts, US) (^1^H-NMR Spectra of the 8 compounds are included in Figure S4).

### 3.3. Mushroom Tyrosinase Inhibition Assay

Tyrosinase inhibitory activity was determined using mushroom tyrosinase (Sigma Aldrich, T3824-250 KU) and 3,4-Dihydroxy-L-phenylalanine (L-DOPA) (Sigma Aldrich, D9628) as enzyme and substrate, respectively. Both were dissolved in 20 mM phosphate buffer solution, pH 6.8. First, 20 µL from a freshly prepared stock solution of mushroom tyrosinase (270 U/mL) and 75 µL of test compounds stock solutions (20 mM PBS, pH 6.8) were mixed and pre-incubated at 37 °C for 15 min. Then, 30 µL of 6 mM L-DOPA was added to the reaction mixture, followed by an incubation of 20 min at 37 °C. During this reaction, L-DOPA is converted to dopachrome (a precursor of melanin), visually detected by the change of color from colorless to orange/brown, resulting in an absorbance increase, recorded spectrophotometrically at 475 nm on a Flex Station 3 Multi-Mode Microplate Reader (Molecular Devices).

The inhibition rate was calculated as follows:

(1) $$\%\;\mathrm{Tyrosinase}\;\mathrm{inhibition}=\left(1-\frac{A-B}{C-D}\right)\times 100$$

where *A* and *B* represent the final and initial optical densities of the reaction in the presence of the tested compounds, while *C* and *D* represent the final and initial optical densities of the reaction in their absence, respectively. Kojic acid (50 μM) was used as positive control. Experiments were carried out in triplicate and repeated at least 3 times.

### 3.4. Kinetic Analysis of Tyrosinase Inhibition

Compounds that exerted the higher inhibitory effect towards tyrosinase activity were further analyzed for their mode of inhibition and inhibition parameters. Initial velocity of the enzymatic reaction, *V*_0_, was determined from the slope of the curve at the initial phase of the reaction, in the presence of increasing concentrations of each compound (25–100 μM) and L-dopa (0.10–1.0 mM) and then plotted as a function of the corresponding substrate concentration, [S]. GraphPad Prism 8.2.1 program was used to fit the obtained data into the Michaelis–Menten model and to determine the kinetic parameters *V*_max_ and *K*_M_ (maximal reaction velocity and Michaelis–Menten constant, respectively). The fits of different enzyme inhibition models (competitive, uncompetitive, noncompetitive, and mixed inhibition) were compared using either Akaike’s Information Criteria (AICc) approach or Extra sum-of-squares *F* test to determine the best fit model for each compound.

### 3.5. Molecular Modeling and Molecular Docking

The crystal structures of mushroom (*Agaricus bisporis*) tyrosinase (mTYR) and human Tyrosinase-related protein 1 (hTYRP1) enzymes complexed with the inhibitor tropolone (PDBID 2Y9X and 5M8O, respectively) [50] were retrieved from the Protein DataBank (http://www.rcsb.org, accessed on 4 April 2020). All crystallographic water and ligand molecules were removed. The PROPKA program [38] was used to check the p*K*_a_ values of all ionizable residues of both enzymes based on their 3D structures. All amino acids preserved the physiological ionization state, except the E98, D336, D353, and E356 of mTYR and H75, E66, H143, and E216 of hTYRP1, which were protonated. The physiological ionization of ligands was used because the enzyme has an optimum pH of 6.8 [46] that is very close to the physiological one, which suggests that the ionization of the ligand compounds should be similar to that in the physiological medium. In addition, the microenvironment of the binding pocket also exhibits several polar interactions between uncharged residues and the docked ligands (e.g., N260, S283, and V283 of mTyr (Figure 4) and N378, T391, and S394 of hTYRP1 (Figure 5)), which also suggests an ionization similar to that observed in solution. GaussView software [51] was used to build the 3D structures of the compounds **1–8** (1 to represent luteolinidin, forms 1-A and 1-C_t_; 2 to represent deoxymalvidin, forms 2-A and 2-C_t_; 3 to represent cyanidin-3-*O*-glucoside, forms 3-B^−^, 3-A^−^, and 3-C_E_^−^; 4 to represent malvidin-3-*O*-glucoside, forms 4-B^−^, 4-A^−^, and 4-C_E_^−^; 5 to represent carboxypyranocyanidin-3-*O*-glucoside; 6 to represent carboxypyranomalvidin-3-*O*-glucoside, forms 6-Q^2−^ and 6-Q^−^; 7 to represent methylpyranocyanidin-3-*O*-glucoside; and 8 to represent methylpyranomalvidin-3-*O*-glucoside).

AutoDock 4.2 software [52] was used for protein:ligand docking calculations. The grid box was centered on the binuclear metal center of both mTYR and hTYRP1 and comprised 56 × 56 × 56 points and 62 × 62 × 62 points, respectively, with a 0.375 Å spacing. The Lamarckian genetic algorithm (LGA) was employed with the following parameters: population size of individuals = 150; maximum number of energy evaluations = 2.5 × 10^6^; and maximum number of generations = 27,000. For all the calculations, 50 docking rounds were performed with step sizes of 2.0 Å for translations and with orientation and torsion step sizes of 5.0°. All docking conformations were clustered within 2.0 Å root-mean-square deviation (RMSd) to prevent similar poses. The Visual Molecular Dynamics (VMD 1.9.2) program [53] was used for visualization of the binding modes, analysis, and image rendering.

### 3.6. Statistical Analysis

Experiments were performed at least three times in triplicates or quadruplicates to ensure the reproducibility of the results. Data are expressed as the mean ± standard error of the mean (SEM). One-way analysis of variance (one-way ANOVA) was used to determine statistically significant differences between the means of different experimental groups using the Tukey’s multiple comparisons test.

## 4. Conclusions

Altogether, the results herein presented suggest that the different anthocyanins and related structures tested have concrete ways of interacting with tyrosinase, and by studying each compound individually, it was possible to draw some conclusions about the effect of different structural features on their modulating effect. Luteolinidin and deoxymalvidin were revealed to be the best performing compounds in the enzymatic inhibition experiments, which was supported by their lower metal coordinating distances and binding energies obtained from the docking simulations, followed by the native anthocyanins and pyrano derivatives.

It must be kept in mind that the stability of anthocyanins is influenced by several factors, such as pH, light, temperature, and complexation with other elements present within the same matrix, such as proteins and other flavonoids, which might compromise their application. Therefore, ensuring their stability remains a very important and challenging issue, though occasionally it seems to be an overlooked topic of discussion. This study, in which different molecular arrangements from a single shared core structure were included, could be a useful starting point for the development of optimized bioinspired inhibitors that reconcile these two crucial aspects—that is, trying to obtain compounds that are structurally more stable without compromising the desired biological effect, or perhaps even enhancing it. Additionally, the different color hues offered by these structurally related compounds broadens the variety of color offer, which could be advantageous from the perspective of the application of natural dyes in the cosmetic industry.

Within the context of dermatological applications for skin-lightening purposes, even considering the good correlation between the in silico results and the enzymatic in vitro inhibition findings, the potential applicability of these compounds must be cautiously addressed, as the mTYR displays several differences from human analogues. Therefore, to consider a straightforward transposition of these results for human-directed applications would be a simplistic and possibly misleading assumption. Nevertheless, the use of the three-dimensional structure of hTYRP1 provided a more robust model, given its homology with hTYR, for predicting binding energies and interactions, instead of solely relying on mushroom-based models. The design of new delivery systems for improving the application of anthocyanins and derivatives in cosmetic formulations should also be considered in the upcoming studies. Considering the outputs of this study, a subsequent evaluation of the effect of these compounds within a set of conditions that resemble in vivo environments using human skin models is intended.

## Figures and Tables

**Table 1:** Inhibition of mushroom tyrosinase by different anthocyanins and related compounds at 50 μM. Superscripted numbers represent the groups (compounds) for which there are statistically significant differences (*p* < 0.0001). Kojic acid was used as positive control. Each experiment was repeated at least 4 times.

Compound	% mTYR Inhibition at 50 µM
Kojic acid **^1−8^**	76.94 ± 3.214
(1) Luteolinidin **^3,5−8^**	58.52 ± 2.082
(3) Cyanidin-3-*O*-glucoside **^2,5−8^**	40.39 ± 1.998
(5) Carboxypyranocyanidin-3-*O*-glucoside **^2,4^**	22.14 ± 0.9620
(7) Methylpyranocyanidin-3-*O*-glucoside **^2,4^**	16.19 ± 1.135
(2) Deoxymalvidin **^6,8^**	57.34 ± 2.381
(4) Malvidin-3-*O*-glucoside **^6^**^,^**^8^**	54.43 ± 4.055
(6) Carboxypyranomalvidin-3-*O*-glucoside	15.71 ± 1.483
(8) Methylpyranomalvidin-3-*O*-glucoside	9.742 ± 3.357

**Table 2:** Kinetic and inhibition constants of mushroom tyrosinase obtained for (1) luteolinidin, (2) deoxymalvidin, (3) cyanidin-3-*O*-glucoside, and (4) malvidin-3-*O*-glucoside.

	IC_50_ (µM)	*K*_i_ (µM)	α	Inhibition Type
Compound **1**	39.21 ± 2.711	15.08 ± 1.182	21.00	Competitive
Compound **2**	20.31 ± 4.308	11.28 ± 0.985	∞	Competitive
Compound **3**	77.91 ± 4.804	40.31 ± 3.611	4.43	Competitive
Compound **4**	27.60 ± 4.202	23.22 ± 1.889	∞	Competitive

**Table 3:** Affinity energy values for the best poses of all compounds into the active site of mTYR, the average distance for the closest Cu-coordinating group, and the most relevant interactions between tyrosinase and all the potential inhibitors. Compounds ranked by their distance to the metal center.

Compound	Δ*G*_binding_ (kcal/mol)	Average Distance to Cu (Å)	Coordinating Group to the Metal	Interactions	
				H-Bonds	Hydrophobic
Luteolinidin (1-A)	−6.0	2.9	O-C7 (ring A)	N260, H263	H263, F264, V283
Luteolinidin (1-C_t_)	−6.6	2.9	HO-C4’ (ring B)	N260, R268	H263, F264, V283
Malvidin-3-*O*-glucoside (4-B^−^)	−8.1	2.9	O-C7 (ring A)	N260, H263	H244, V248, H263, F264, V283
Cyanidin-3-*O*-glucoside (3-A^−^)	−8.5	2.9	O-C7 (ring A)	H263, R268, S282	V248, N260, H263, F264, V283
Malvidin-3-*O*-glucoside (3-A^−^)	−8.4	2.9	O-C7 (ring A)	H244, M280, G281, S282	V248, H263, F264, V283, A286
Deoxymalvidin (2-C_t_)	−7.0	3.0	O-C7 (ring A)	H244, H263, N260	H263, F264, V283
Deoxymalvidin (2-A)	−6.0	3.0	O-C7 (ring A)	N260, H263	H244, H263, F264, V283
Cyanidin-3-*O*-glucoside (3-B^−^)	−9.0	3.1	O-C7 (ring A)	H263, S282	V248, H263, F264, V283
Kojic acid (KA)	−4.5	3.3	OH	N260	H263, F264, V283
Tropolone	−4.9	3.4	O	-	H263, F264, V283
Malvidin-3-*O*-glucoside (4-C_E_^−^)	−8.6	3.5	O-C7 (ring A)	H263 R268, S282	H244, V248, H263, F264, V283
Cyanidin-3-*O*-glucoside (3-C_E_^−^)	−8.8	3.5	O-C7 (ring A)	N260, R268	H263, F264, V283
Carboxypyranomalvidin-3-*O*-glucoside (6-A^2−^)	−8.3	3.6	O-C4’ (ring B)	H244, R268, S282	V248, F264, V283
Methylpyranocyanidin-3-*O*-glucoside (7)	−8.6	3.6	O-C4’ (ring B)	H244, R268, S282	V248, F264, V283
Carboxypyranomalvidin-3-*O*-glucoside (6-A^−^)	−7.9	3.6	OH-C4’ (ring B)	H244, R268, S282	V248, F264, V283
Carboxypyranocyanidin-3-*O*-glucoside (5)	−6.9	3.8	O-C4’ (ring B)	H244, N260, R268	V248, F264, V283
Methylpyranomalvidin-3-*O*-glucoside (8)	−8.8	4.5	O-C7 (ring A)	R268, S282	V248, F264, V283

**Table 4:** Affinity energy values for the best poses of all compounds into the active site of hTYRP1, the average distance for the closest Zn-coordinating group, and the most relevant interactions between tyrosinase and all the potential inhibitors. Compounds ranked by their distance to the metal center.

Compound	Δ*G*_binding_ (kcal/mol)	Average Distance to Zn (Å)	Coordinating Group to the Metal	Interactions	
				H-bonds	Hydrophobic
Luteolinidin (1-C_t_)	−7.1	1.9	HO-C3’, HO-C4’ (ring B)	Y362, S394	Y362, H381
Tropolone	−6.0	2.0	O	T391, S394	L382, H381, T391
Kojic acid	−5.2	2.0	O	S394	L382, H381, T391
Luteolinidin (1-A)	−7.5	2.1	HO-C3’, HO-C4’ (ring B)	R374, S394	Y362, L382, H381
Malvidin-3-*O*-glucoside (4-B^−^)	−7.6	2.3	O-C7 (ring A)	R374, G389, T391	Y362, L382, H381
Deoxymalvidin (2-C_t_)	−6.8	2.4	HO-C4’ (ring B)	Y362, S394	H381
Methylpyranocyanidin-3-*O*-glucoside (7)	−8.6	2.4	O-C7 (ring A)	Y362, T391, S394	Y362, L382, H381
Deoxymalvidin (2-A)	−6.1	2.5	O-C7 (ring A)	N378, T391, S394	L382, H381
Cyanidin-3-*O*-glucoside (3-B^−^)	−10.8	2.6	O-C7 (ring A)	Y362, R374, N378, T391, S394	Y362, L382, H381
Cyanidin-3-*O*-glucoside (3-A^−^)	−8.5	2.6	O-C7 (ring A)	R321, R354, H377, N378, T391, S394	L382, H381, T391, F400
Malvidin-3-*O*-glucoside (3-A^−^)	−8.4	2.7	O-C7 (ring A)	R321, Y362, R374, G388, G389, T391	L382, H381, Q390
Carboxypyranomalvidin-3-*O*-glucoside (6-A^2−^)	−9.6	2.7	COO- (ring D)	D212, E216, Y362, R374, T391	L382
Carboxypyranocyanidin-3-*O*-glucoside (5)	−9.4	2.9	COO- (ring D)	N378, T391, S394	Y362, L382
Carboxypyranomalvidin-3-*O*-glucoside (6-A^−^)	−10.0	2.9	COO- (ring D)	D212, E216, R374, T391	Y362
Methylpyranomalvidin-3-*O*-glucoside (8)	−8.7	3.2	OMe-C3’ (ring B)	Y362, R374, N378, T391	L382
Cyanidin-3-*O*-glucoside (3-Cc^−^)	−7.3	4.2	O-C7 (ring A)	R374, G389, T391	L382
Malvidin-3-*O*-glucoside (4-Cc^−^)	−9.4	5.6	O-C7 (ring A)	R374, T391	L382

## Data Availability

Not applicable.

## Supplementary Materials

Supplementary materials can be found at https://www.mdpi.com/article/10.3390/ijms22126192/s1.
