# Supplementary material for: The Role of Anthocyanins, Deoxyanthocyanins and Pyranoanthocyanins on the Modulation of Tyrosinase Activity: An In Vitro and In Silico Approach
Source: Int J Mol Sci. 2021 Jun 8;22(12):6192. doi: 10.3390/ijms22126192 (PMC8230073; doi:10.3390/ijms22126192)
Supplement: Supplementary file 1 [file ijms-22-06192-s001.zip › ijms-1241351-supplementary.pdf]

# Supplementary Information

# The Role of Anthocyanins, Deoxyanthocyanins and Pyranoanthocyanins on the Modulation of Tyrosinase Activity: An In Vitro and In Silico Approach

**Patrícia Correia [1,†], Hélder Oliveira [1,†], Paula Araújo [1], Natércia F. Brás [1], Ana Rita Pereira [1], Joana Moreira [2,3], Victor de Freitas [1], Nuno Mateus [1], Joana Oliveira [1,*] and Iva Fernandes [1,*]**

[1] LAQV-REQUIMTE, Department of Chemistry and Biochemistry, Faculty of Sciences, University of Porto, 4169-007 Porto, Portugal; patricia.correia@fc.up.pt (P.C.); helder.oliveira@fc.up.pt (H.O.); paula.araujo@fc.up.pt (P.A.); nbras@fc.up.pt (N.F.B.); anarita@fc.up.pt (A.R.P.); vfreitas@fc.up.pt (V.d.F.); nbmateus@fc.up.pt (N.M.)

[2] Laboratório de Química Orgânica e Farmacêutica, Departamento de Ciências Químicas, Faculty of Pharmacy of the University of Porto, Rua Jorge Viterbo Ferreira No. 228, 4050-313 Porto, Portugal; up201302558@edu.ff.up.pt

[3] Centro Interdisciplinar de Investigação Marinha e Ambiental (CIIMAR), Universidade do Porto, Edifício do Terminal de Cruzeiros do Porto de Leixões, Av. General Norton de Matos s/n, 4050-208 Matosinhos, Portugal

* Correspondence: jsoliveira@fc.up.pt (J.O.); iva.fernandes@fc.up.pt (I.F.)

† Both authors equally contributed to the work

**Table S1.** Best-fit values of the kinetic constants of mTYR and its natural substrate L-DOPA, in the presence of increasing concentrations of luteolinidin, deoxymalvidin, cyanidin-3-*O*-glucoside and malvidin-3-*O*-glucoside obtained from GraphPad Prism 8.2.1 program analysis. Maximal velocity was expressed as micromoles of product formed per minute at 475 nm, considering the molar absorptivity value of L-dopachrome (3600 $M^{-1}$ $cm^{-1}$). Parameters for which the program was unable to calculate a complete confidence interval are represented as "/". Each experiment was repeated 3 times.

| Inhibitor (concentration, μM) | $K_m$ ($K_m'$) ± SEM (mM) | $V_{max}$ ± SEM (μM/min) |
|---|---|---|
| - | 0.57 ± 0.07 | 46.3 ± 2.9 |
| Luteolinidin (25) | 0.86 ± 0.26 | 45.8 ± 2.2 |
| Luteolinidin (50) | 1.36 ± 0.61 | 45.8 ± 2.2 |
| Luteolinidin (75) | 2.39 ± 1.43 | 45.8 ± 2.2 |
| Luteolinidin (100) | / | 45.8 ± 2.2 |
| Deoxymalvidin (25) | 7.29 ± 1.26 | 46.4 ± 2.4 |
| Deoxymalvidin (50) | / | 46.4 ± 2.4 |
| Deoxymalvidin (75) | 1.86 ± 0.14 | 46.4 ± 2.4 |
| Deoxymalvidin (100) | 2.06 ± 0.93 | 46.4 ± 2.4 |
| Cyanidin-3-*O*-glucoside (25) | 0.78 ± 0.17 | 45.1 ± 2.2 |
| Cyanidin-3-*O*-glucoside (50) | 0.87 ± 0.16 | 45.1 ± 2.2 |
| Cyanidin-3-*O*-glucoside (75) | 0.90 ± 0.11 | 45.1 ± 2.2 |
| Cyanidin-3-*O*-glucoside (100) | 1.03 ± 0.23 | 45.1 ± 2.2 |
| Malvidin-3-*O*-glucoside (25) | 1.21 ± 0.46 | 46.1 ± 2.3 |
| Malvidin-3-*O*-glucoside (50) | 3.56 ± 2.60 | 46.1 ± 2.3 |
| Malvidin-3-*O*-glucoside (75) | / | 46.1 ± 2.3 |
| Malvidin-3-*O*-glucoside (100) | 7.53 ± 2.62 | 46.1 ± 2.3 |

**Table S2**. Affinity energy values for different binding poses of all compounds against the active site of *m*TYR as well as the distances between the closest coordinating group to the metal center.

| Compound | $\Delta G_{\text{binding}}$ (kcal/mol) | Distance (Å) | | | Coordinating group to metal |
|---|---|---|---|---|---|
| | | CuA | CuB | average | |
| Tropolone | -4.9 | 3.7 | 3.1 | 3.4 | O |
| Kojic acid (KA) | -4.5 | 3.2 | 3.5 | 3.3 | OH |
| Luteolinidin (1-$C_t$) | -7.0 | 3.7 | 3.2 | 3.5 | O-C7 (ring A) |
| Luteolinidin (1-$C_t$) | -6.6 | 3.0 | 2.8 | 2.9 | HO-C4' (ring B) |
| Luteolinidin (1-A) | -6.4 | 7.1 | 4.8 | 5.9 | HO-C3', HO-C4' (ring B) |
| Luteolinidin (1-A) | -6.0 | 2.9 | 2.9 | 2.9 | O-C7 (ring A) |
| Deoxymalvidin (2-$C_t$) | -7.0 | 3.0 | 2.9 | 3.0 | O-C7 (ring A) |
| Deoxymalvidin (2-A) | -6.6 | 6.4 | 4.7 | 5.5 | OMe-C3' (ring B) |
| Deoxymalvidin (2-A) | -6.0 | 2.8 | 3.2 | 3.0 | O-C7 (ring A) |
| Deoxymalvidin (2-$C_t$) | -5.5 | 3.2 | 2.7 | 3.0 | HO-C4' (ring B) |
| Cyanidin-3-*O*-glucoside (3-$B^-$) | -9.0 | 3.4 | 2.8 | 3.1 | O-C7 (ring A) |
| Cyanidin-3-*O*-glucoside (3-$C_c^-$) | -8.8 | 3.9 | 3.2 | 3.5 | O-C7 (ring A) |
| Cyanidin-3-*O*-glucoside (3-$C_c^-$) | -8.8 | 4.4 | 3.0 | 3.7 | HO-C4' (ring B) |
| Cyanidin-3-*O*-glucoside (3-$B^-$) | -8.2 | 4.0 | 3.3 | 3.7 | HO-C3', HO-C4' (ring B) |
| Malvidin-3-*O*-glucoside (4-$B^-$) | -9.8 | 3.7 | 3.2 | 3.4 | HO-C4' (ring B) |
| Malvidin-3-*O*-glucoside (4-$C_c^-$) | -9.1 | 5.4 | 4.0 | 4.7 | HO-C4', OMe-C3' (ring B) |
| Malvidin-3-*O*-glucoside (4-$C_c^-$) | -8.6 | 3.9 | 3.1 | 3.5 | O-C7 (ring A) |
| Malvidin-3-*O*-glucoside (4-$B^-$) | -8.1 | 2.9 | 2.9 | 2.9 | O-C7 (ring A) |
| Carboxypyranocyanidin-3-*O*-glucoside (5) | -7.3 | 6.9 | 4.6 | 5.7 | O-C7 (ring A) |
| Carboxypyranocyanidin-3-*O*-glucoside (5) | -6.9 | 4.0 | 3.7 | 3.8 | O-C4' (ring B) |
| Carboxypyranocyanidin-3-*O*-glucoside (5) | -6.8 | 5.0 | 4.2 | 4.6 | COO- (ring D) |
| Carboxypyranomalvidin-3-*O*-glucoside (6-$A^-$) | -8.3 | 4.0 | 4.3 | 4.2 | COO- (ring D) |
| Carboxypyranomalvidin-3-*O*-glucoside (6-$A^-$) | -7.9 | 3.8 | 3.4 | 3.6 | HO-C4' (ring B) |
| Carboxypyranomalvidin-3-*O*-glucoside (6-$A^-$) | -7.2 | 5.5 | 4.9 | 5.2 | O-C7 (ring A) |
| Carboxypyranomalvidin-3-*O*-glucoside (6-$A^{2-}$) | -8.3 | 3.8 | 3.4 | 3.6 | O-C4' (ring B) |
| Carboxypyranomalvidin-3-*O*-glucoside (6-$A^{2-}$) | -7.2 | 5.8 | 3.9 | 4.9 | O-C7 (ring A) |
| Carboxypyranomalvidin-3-*O*-glucoside (6-$A^{2-}$) | -7.1 | 5.4 | 5.5 | 5.4 | COO- (ring D) |
| Methylpyranocyanidin-3-*O*-glucoside (7) | -8.8 | 4.6 | 3.9 | 4.3 | O-C7 (ring A) |
| Methylpyranocyanidin-3-*O*-glucoside (7) | -8.6 | 4.4 | 2.8 | 3.6 | O-C4' (ring B) |
| Methylpyranomalvidin-3-*O*-glucoside (8) | -8.8 | 5.4 | 3.6 | 4.5 | O-C7 (ring A) |
| Methylpyranomalvidin-3-*O*-glucoside (8) | -8.5 | 6.9 | 5.4 | 6.2 | HO-C4', OMe-C3' (ring B) |

**Table S3**. Affinity energy values for different binding poses of all compounds against the active site of *h*TYRP1 as well as the distances between the closest coordinating group to the metal center.

| Compound | $\Delta G_{\text{binding}}$ (kcal/mol) | Distance (Å) | | | Coordinating group to metal |
|---|---|---|---|---|---|
| | | ZnA | ZnB | average | |
| Tropolone | -6.0 | 1.8 | 2.2 | 2.0 | O |
| Kojic acid (KA) | -5.2 | 1.9 | 2.1 | 2.0 | O |
| Luteolinidin (1-$C_t$) | -8.5 | 2.3 | 3.1 | 2.7 | CO |
| Luteolinidin (1-$C_t$) | -7.6 | 1.7 | 2.7 | 2.2 | HO-C7 (ring A) |
| Luteolinidin (1-A) | -7.5 | 1.7 | 2.5 | 2.1 | HO-C3', HO-C4' (ring B) |
| Luteolinidin (1-$C_t$) | -7.1 | 1.9 | 2.0 | 1.9 | HO-C3', HO-C4' (ring B) |
| Deoxymalvidin (2-A) | -6.9 | 1.7 | 3.3 | 2.5 | HO-C5 (ring A) |
| Deoxymalvidin (2-A) | -6.5 | 1.9 | 3.2 | 2.5 | HO-C4' (ring B) |
| Deoxymalvidin (2-A) | -6.1 | 1.9 | 3.2 | 2.5 | O-C7 (ring A) |

| | | | | | |
|---|---|---|---|---|---|
| Deoxymalvidin (2-$C_t$) | -6.8 | 2.0 | 2.7 | 2.4 | HO-C4' (ring B) |
| Deoxymalvidin (2-$C_t$) | -6.4 | 1.8 | 4.1 | 2.9 | O-C7 (ring A) |
| Cyanidin-3-*O*-glucoside (3-$B^-$) | -10.8 | 1.8 | 3.5 | 2.6 | O-C7 (ring A) |
| Cyanidin-3-*O*-glucoside (3-$C_c^-$) | -7.3 | 4.2 | 4.3 | 4.2 | O-C7 (ring A) |
| Malvidin-3-*O*-glucoside (4-$B^-$) | -7.6 | 1.7 | 3.0 | 2.3 | O-C7 (ring A) |
| Malvidin-3-*O*-glucoside (4-$C_c^-$) | -9.4 | 5.5 | 5.7 | 5.6 | O-C7 (ring A) |
| Carboxypyranocyanidin-3-*O*-glucoside (5) | -10.7 | 4.1 | 4.3 | 4.2 | O-C7 (ring A) |
| Carboxypyranocyanidin-3-*O*-glucoside (5) | -9.4 | 2.0 | 3.8 | 2.9 | COO- (ring D) |
| Carboxypyranomalvidin-3-*O*-glucoside (6-$A^-$) | -10.7 | 4.3 | 4.4 | 4.4 | O-C7 (ring A) |
| Carboxypyranomalvidin-3-*O*-glucoside (6-$A^-$) | -10.0 | 2.4 | 3.5 | 2.9 | COO- (ring D) |
| Carboxypyranomalvidin-3-*O*-glucoside (6-$A^{2-}$) | -9.9 | 4.1 | 4.1 | 4.1 | O-C7 (ring A) |
| Carboxypyranomalvidin-3-*O*-glucoside (6-$A^{2-}$) | -9.6 | 2.4 | 3.0 | 2.7 | COO- (ring D) |
| Methylpyranocyanidin-3-*O*-glucoside (7) | -8.6 | 1.9 | 2.9 | 2.4 | O-C7 (ring A) |
| Methylpyranocyanidin-3-*O*-glucoside (7) | -8.1 | 3.1 | 3.6 | 3.4 | O-C4' (ring B) |
| Methylpyranomalvidin-3-*O*-glucoside (8) | -8.7 | 2.4 | 4.0 | 3.2 | OMe-C3' (ring B) |
| Methylpyranomalvidin-3-*O*-glucoside (8) | -8.3 | 4.4 | 4.6 | 4.5 | O-C7 (ring A) |

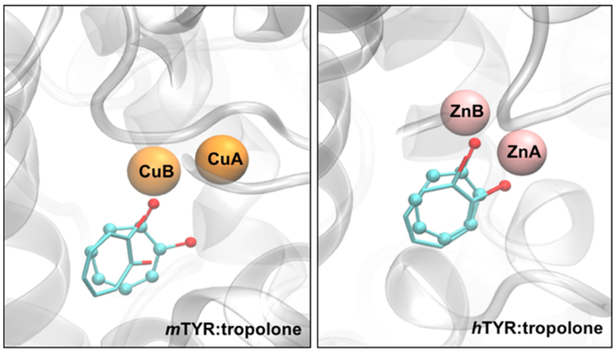


**Figure S1.** Superposition of the crystallographic tropolone (depicted in sticks) with the respective top-ranked docking pose (depicted in balls-and-sticks). The copper and zinc ions are depicted in van der Waals (vdW) and colored in orange and pink, respectively; the tyrosinase is represented in cartoon and colored in gray.

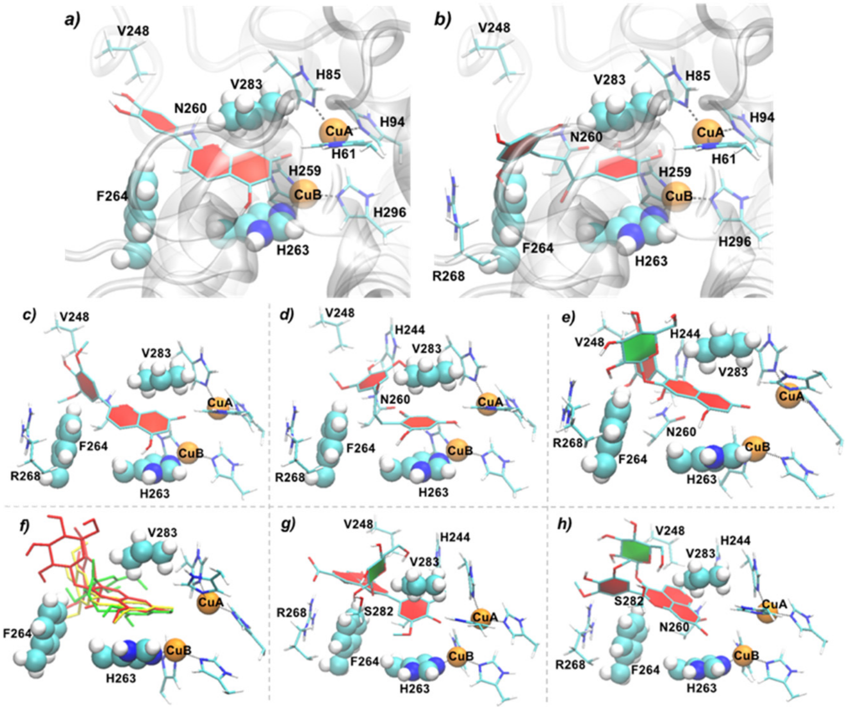


**Figure S2**. 3D representation of the best docking pose of some representative compounds toward the active site of mTYR: a) 1-A, b) 1-Ct, c) 2-A, d) 2-Ct, e) 4-B$^-$, f) superposition of 2-A (yellow), 2Ct (green) and 4-B$^-$ (red), g) 6, and h) 8. Compounds are represented with sticks and colored by atom type; the copper ions are depicted in van der Waals (VdW) and colored in orange; the tyrosinase is represented in cartoon and colored in gray, while the most interacting residues are represented in sticks (except H263, F264 and V283 shown in vdW) and colored by atom type. Aromatic and non-aromatic rings of the compounds are colored in red and green, respectively.

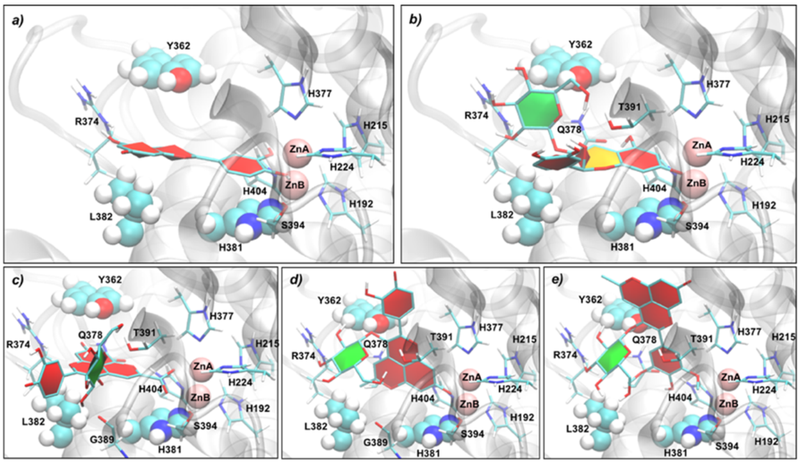


**Figure S3**. 3D representation of the best docking pose of some representative compounds toward the active site of hTYRP1: a) 1-A, b) 3-B-, c) 5, d) 7, and e) 8. Compounds are represented with sticks and colored by atom type; zinc ions are depicted in van der Waals (vdW) and colored in pink; tyrosinase is represented in cartoon and colored in gray, while the most interacting residues are represented in sticks (except H381, Y362 and L382 shown in vdW) and colored by atom type. Aromatic, non-aromatic saccharide and non-aromatic non-saccharide rings of the compounds are colored in red, green and orange, respectively.

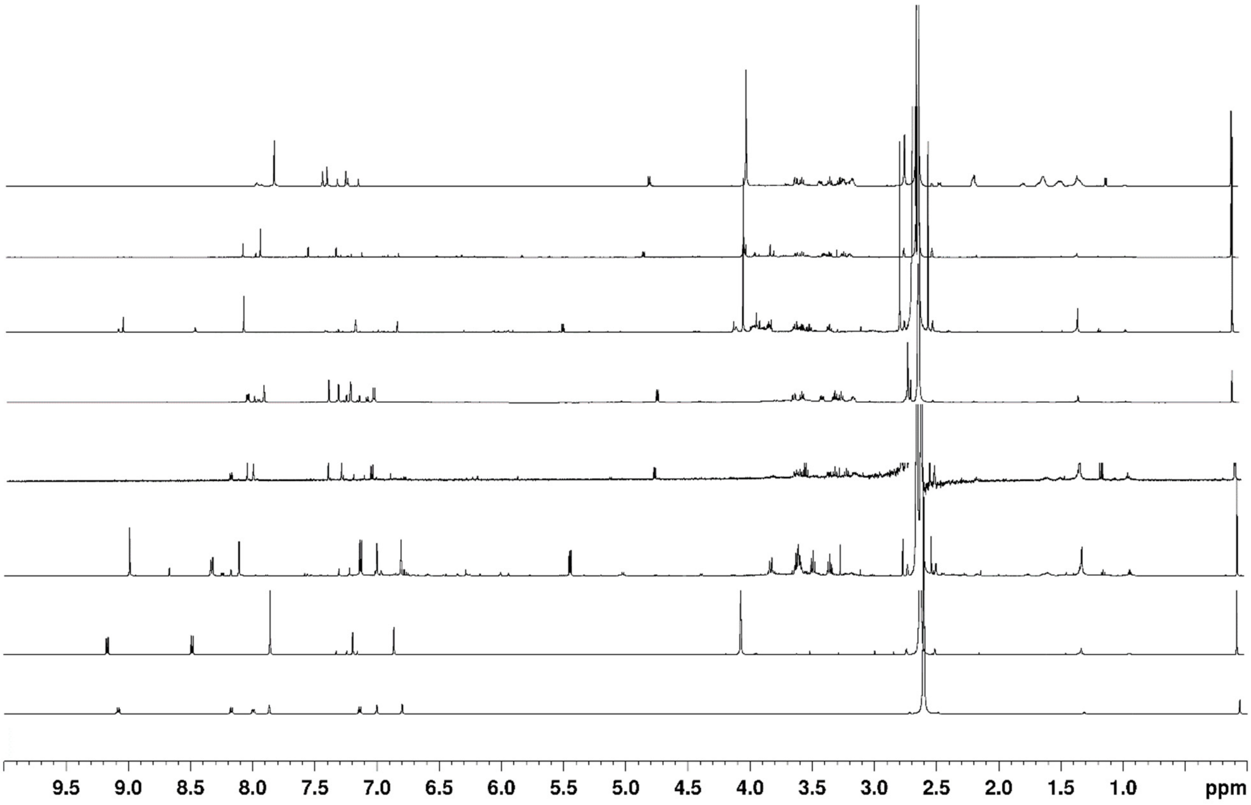


**Figure S4.** $^{1}$H-NMR (600.13 MHz) spectrum of luteolinidin, deoxymalvidin, cyanidin-3-*O*-glucoside, carboxypyranocyanidin-3-*O*-glucoside, methylpyranocyanidin-3-*O*-glucoside, malvidin-3-*O*-glucoside, carboxypyranomalvidin-3-*O*-glucoside and methylpyranomalvidin-3-*O*-glucoside (from bottom to top), at 25 ºC in $(CD_3)_2S{=}O$ /TFA (90:10, v/v).
